# Differential Modulation of Ventral Tegmental Area Circuits by the Nociceptin/Orphanin FQ System

**DOI:** 10.1523/ENEURO.0376-19.2020

**Published:** 2020-09-23

**Authors:** Joseph R. Driscoll, Tanya L. Wallace, Kasra A. Mansourian, William J. Martin, Elyssa B. Margolis

**Affiliations:** 1 BlackThorn Therapeutics, San Francisco, CA 94103; 2 UCSF Weill Institute of Neurosciences, Department of Neurology, University of California, San Francisco, San Francisco, CA 94143

**Keywords:** dopamine, nociceptin, orphanin FQ, ventral tegmental area, voltage clamp, voltammetry

## Abstract

The neuropeptide nociceptin/orphanin FQ (N/OFQ) can be released by stressors and is associated with disorders of emotion regulation and reward processing. N/OFQ and its receptor, NOP, are enriched in dopaminergic pathways, and intra-ventricular agonist delivery decreases dopamine levels in the dorsal striatum, nucleus accumbens (NAc), and ventral tegmental area (VTA). We used whole-cell electrophysiology in acute rat midbrain slices to investigate synaptic actions of N/OFQ. N/OFQ was primarily inhibitory, causing outward currents in both immunocytochemically identified dopaminergic (tyrosine hydroxylase positive (TH(+))) and non-dopaminergic (TH(–)) VTA neurons; effect at 1 μm: 20 ± 4 pA. Surprisingly, this effect was mediated by augmentation of postsynaptic GABA_A_R currents, unlike the substantia nigra pars compacta (SNc), where the N/OFQ-induced outward currents were K^+^ channel dependent. A smaller population, 17% of all VTA neurons, responded to low concentrations of N/OFQ with inward currents (10 nm: −11 ± 2 pA). Following 100 nm N/OFQ, the response to a second N/OFQ application was markedly diminished in VTA neurons (14 ± 10% of first response) but not in SNc neurons (90 ± 20% of first response). N/OFQ generated outward currents in medial prefrontal cortex (mPFC)-projecting VTA neurons, but inward currents in a subset of posterior anterior cingulate cortex (pACC)-projecting VTA neurons. While N/OFQ inhibited NAc-projecting VTA cell bodies, it had little effect on electrically or optogenetically evoked terminal dopamine release in the NAc measured *ex vivo* with fast scan cyclic voltammetry (FSCV). These results extend our understanding of the N/OFQ system in brainstem circuits implicated in many neurobehavioral disorders.

## Significance Statement

The neuropeptide nociceptin/orphanin FQ (N/OFQ) and its receptor (NOP) are engaged under conditions of stress and are associated with reward processing disorders. Both peptide and receptor are highly enriched in ventral tegmental area (VTA) pathways underlying motivation and reward. Using whole-cell electrophysiology in rat midbrain slices we found: (1) NOPs are functional on both dopaminergic and non-dopaminergic VTA neurons; (2) N/OFQ differentially regulates VTA neurons based on neuroanatomical projection target; and (3) repeated application of N/OFQ produces evidence of receptor desensitization in VTA but not SNc neurons. These results reveal candidate mechanisms by which the NOP system regulates motivation and emotion.

## Introduction

Nociceptin/orphanin FQ (N/OFQ) and its receptor (NOP) make up a neuropeptide signaling system de-orphaned in 1995 (Meunier et al., 1995; Reinscheid et al., 1995) that is engaged under conditions of stress (Ciccocioppo et al., 2000; Devine et al., 2001; Nicholson et al., 2002; Fernandez et al., 2004; Leggett et al., 2006, 2007; Green et al., 2007; Green and Devine, 2009; Nativio et al., 2012). The NOP is a G-protein coupled seven-transmembrane domain receptor that canonically signals through Gi/o proteins, postsynaptically activating G-protein-coupled inward-rectifying potassium channels (GIRKs), or presynaptically reducing probability of neurotransmitter release via inhibition of N-type calcium channels (Knoflach et al., 1996; Vaughan and Christie, 1996; Hawes et al., 2000; New and Wong, 2002). While amino acid sequence homology has led some to categorize the NOP as an opioid receptor (Bunzow et al., 1994; Mollereau et al., 1994; Wang et al., 1994; Meunier et al., 1995), NOP activation is not blocked by naloxone, a non-selective opioid receptor antagonist that was originally used to classify responses as opioid receptor mediated, blocking activation at μ, δ, and κ opioid receptors (MOPs, DOPs, and KOPs, respectively; Reinscheid et al., 1995, 1996; Gintzler et al., 1997; Mogil and Pasternak, 2001). Furthermore, the known endogenous opioid peptides (dynorphins, enkephalins, and endorphins) do not bind to the NOP, and N/OFQ does not bind to the MOP, DOP, or KOP (Meng et al., 1996; Sim et al., 1996; Ma et al., 1997). Because of the extensive amino acid sequence homology and these distinct pharmacological properties, N/OFQ and the NOP are most appropriately subclassified as non-classical members of the opioid family (Cox et al., 2015; Toll et al., 2016).

N/OFQ and the NOP are highly enriched in the ventral tegmental area (VTA), dorsal striatum, nucleus accumbens (NAc), medial prefrontal cortex (mPFC), and central nucleus of the amygdala (Neal et al., 1999; Berthele et al., 2003; Parker et al., 2019). The VTA is the major source of dopamine to limbic forebrain regions and plays a key role in brain networks that coordinate motivation and learned appetitive behaviors (Fields et al., 2007). Activity of VTA dopamine neurons is associated with salience and reward prediction, while destruction of these neurons results in motivational deficits (Ungerstedt, 1971; Wise, 2005; Fields et al., 2007; Tsai et al., 2009; Witten et al., 2011; Kim et al., 2012; Morales and Margolis, 2017; Mohebi et al., 2019). Intracerebroventricular (ICV) injections of N/OFQ produce a decrease in extracellular dopamine in the dorsal striatum and NAc, and some midbrain putative dopamine cell bodies are inhibited by NOP activation (Murphy et al., 1996; Murphy and Maidment, 1999; Di Giannuario and Pieretti, 2000; Lutfy et al., 2001; Zheng et al., 2002; Vazquez-DeRose et al., 2013).

Dysregulation of the N/OFQ system has been associated with disorders of motivated responding (Civelli and Zhou, 2008), and the N/OFQ system has been investigated as a novel therapeutic target for major depressive disorder and alcohol use disorder (Witkin et al., 2019); however, understanding the involvement of the N/OFQ system in these behaviors remains a challenge. In fact, in some cases, activation and blockade of NOPs paradoxically produce the same behavioral outcomes, for example with alcohol consumption (Kuzmin et al., 2007; Ciccocioppo et al., 2014; Rorick-Kehn et al., 2016) and anxiety-related behaviors (Jenck et al., 1997; Dautzenberg et al., 2001; Gavioli et al., 2002; Fernandez et al., 2004; Kamei et al., 2004; Vitale et al., 2006; Green et al., 2007; Varty et al., 2008). Such observations may be explained by off-target effects of N/OFQ, activation of N/OFQ sensitive neural circuits that compete for behavioral control, or receptor desensitization.

Here, we investigated the basic physiology of N/OFQ responses in VTA neurons to better characterize how N/OFQ contributes to motivation and reward processing. To confirm that our physiological responses to N/OFQ were because of NOP activation we used the selective NOP antagonist BTRX-246040 (Toledo et al., 2014) to block N/OFQ responses. We observed similar N/OFQ effects on both dopamine and non-dopamine VTA neurons. Importantly, we found that responses to N/OFQ differ between VTA and substantia nigra pars compacta (SNc) neurons in mechanism of inhibition and functional desensitization measures. Furthermore, we found that for VTA neurons, N/OFQ responses vary by the projection target. For example, N/OFQ induced small inward currents preferentially in VTA neurons that project to the posterior anterior cingulate cortex (pACC). In addition, although NAc-projecting cell bodies were inhibited by NOP activation, N/OFQ induced minimal inhibition of dopamine release at terminals in the NAc. Together, these observations indicate that NOP actions vary not only by brain region and neuron subpopulation but also by structural localization within a neuron.

## Materials and Methods

### Electrophysiology

Most experiments were completed in tissue from male Sprague Dawley rats, post-natal day 22–36, except mechanism experiments which were completed in tissue from adult rats (>200 g). Rats were anesthetized with isoflurane, and brains were removed. The brains were submerged in Ringer’s solution containing the following: 119 mm NaCl, 2.5 mm KCl, 1.3 mm MgSO_4_, 1.0 mm NaH_2_PO_4_, 2.5 mm CaCl_2_, 26.2 mm NaHCO_3_, and 11 mm glucose saturated with 95% O_2_–5% CO_2_ and horizontal brain slices (150 µm thick) containing the VTA were prepared using a Vibratome (Leica Instruments). Slices were then allowed to recover at 35°C for at least 1 h before recordings were initiated. The same Ringer’s solution was used for cutting, recovery, and recording.

Individual slices were visualized under an Olympus BX50WI microscope (Olympus Life Science Solutions) with differential interference contrast optics and near infrared illumination, using an Andor xIon+ camera, and Andor Solis imaging software (Andor Technology Ltd), or under a Zeiss Axio Examiner.D1 with differential interference contrast optics, near infrared illumination, and Dodt contrast, using a monochrome Axiocam 506 (Zeiss International). Whole-cell patch-clamp recordings were made at 33°C using 2.5–4 MΩ pipettes containing the following: 123 mm K-gluconate, 10 mm HEPES, 0.2 mm EGTA, 8 mm NaCl, 2 mm MgATP, and 0.3 mm Na_3_GTP, pH 7.2, osmolarity adjusted to 275 mOsm. Biocytin (0.1%) was added to the internal solution for *post hoc* identification.

Recordings were made using an Axopatch 1-D (Molecular Devices), filtered at 2 kHz, and collected at 20 kHz using IGOR Pro (Wavemetrics) or an IPA amplifier with SutterPatch software (Sutter Instrument) filtered at 1 kHz and collected at 10 kHz. Liquid junction potentials were not corrected during recordings. Hyperpolarization-activated cation currents (*I*_h_) were recorded by voltage clamping cells and stepping from −60 to −40, −50, −70, −80, −90, −100, −110, and −120 mV. The *I*_h_ magnitude was measured as the difference between the initial response to the voltage step after the capacitive peak and the final current response.

Pharmacology experiments were completed in voltage-clamp mode (V = −60 mV) to measure changes in membrane current. Series resistance was monitored online by measuring the peak of the capacitance transient in response to a −4 mV voltage step applied at the onset of each sweep. Input resistance was measured using the steady state response to the same voltage step. Upon breaking into the cell, at least 10 min was allowed for the cell to stabilize and for the pipette internal solution to dialyze into the cell. Drugs were applied via bath perfusion at a flow rate of 2 mL/min or pressure ejection using a SmartSquirt micro-perfusion system (AutoMate Scientific) coupled to a 250-µm inner diameter tubing outlet positioned nearby the recorded cell (within ∼200 µm). N/OFQ (1 nm to 10 μm) was bath applied (5–7 min) or pressure injected (2 min) only after a 5-min stable baseline was achieved. Responses were similar to the two forms of N/OFQ application at the same concentrations. For instance, at 100 nm, bath application 10.1 ± 1.5 pA, *n* = 21; pressure ejection 9.8 ± 2.1 pA, *n* = 12. As there was no statistical difference in the mean amplitude of response for bath application and pressure injection, the results were combined for the analysis. Any cell that showed drift or did not maintain a consistent baseline current for the full 5-min period was removed from the analysis. All experiments where repeated N/OFQ applications are reported, such as the desensitization experiments, were completed with bath application. To test that observed N/OFQ-mediated effects were specific to NOP, the selective NOP antagonist BTRX-246040 (100 nm) was applied for 10 min before N/OFQ.

For iontophoresis experiments, the holding current was set to −50 mV to increase the driving potential for Cl^–^. GABA (100 mm, pH adjusted to 4.9 with 37% HCl) was prepared daily and the GABA-containing pipette was positioned ∼50 µm away from the recorded neuron. Negative retention current (approximately −35 nA) was applied to the GABA pipette, interrupted by positive ejection current pulses (100 ms) once every 30 s, with the intensity adjusted so that the response amplitude was in the range of 100–300 pA.

Stock solutions of drugs were made in advance, stored at −20°C, and diluted into artificial CSF (aCSF) immediately before application. N/OFQ was obtained from Tocris and diluted to a 100 μm stock solution in ddH_2_O. Stock BTRX-246040 was obtained from BlackThorn Therapeutics and dissolved in dimethylsulfoxide (10 mm).

### Retrograde tracer injections

Male Sprague Dawley rats, 21–100 d old, were anesthetized with isoflurane. A glass pipette (30- to 50-µm tip) connected to a Nanoject II/Nanoliter 2000 microinjector (Drummond Scientific Co) was stereotaxically placed in the mPFC [from bregma: anteroposterior (AP), +2.6 mm; mediolateral (ML), ±0.8 mm; ventral (DV), −4.0 mm from skull surface], the pACC (AP, 1.6 mm; ML, ±0.6 mm; V, −3.5 mm), or the NAc (AP, +1.5 mm; ML, ±0.8 mm; V, −6.7 mm). Neuro-DiI (7% in ethanol; Biotium) was slowly injected, 50.6 nL per side. Animals were allowed to recover for 5–7 d, while the retrograde tracer transported back to the cell bodies. On the day of recording, the experimenter was blind to the location of retrograde tracer injection (mPFC, pACC, or NAc), and slices were prepared as above. Projection neurons were chosen by selecting cells observed as labeled using epifluorescent illumination. All injection sites were histologically confirmed by a third party blind to the electrophysiology results to avoid bias. N/OFQ responses were analyzed before unblinding. Animals with improper injection placements or significant diffusion outside of the target region were rejected.

### Immunohistochemistry

Slices were preblocked for 2 h at room temperature in PBS with 0.2% BSA and 5% normal goat serum, then incubated at 4°C with a rabbit anti-TH polyclonal antibody (1:100; EMD Millipore, RRID: AB_390204). Slices were then washed thoroughly in PBS with 0.2% BSA before being agitated overnight at 4°C with Cy5 anti-rabbit secondary antibody (1:100; Jackson ImmunoResearch, RRID: AB_2534032) and FITC streptavidin (6.5 µL/ml). Sections were rinsed and mounted on slides using Bio-Rad Fluoroguard Antifade Reagent mounting media and visualized with an Axioskop FS2 Plus microscope with an Axiocam MRm running Neurolucida (MBF Biosciences). Neurons were only considered TH(−) if there was no colocalization of biocytin with TH signal and the biocytin soma was in the same focal plane as other TH(+) cell bodies. Primary antibodies were obtained from Millipore Bioscience Research Reagents or Millipore, secondary antibodies were obtained from Jackson ImmunoResearch, and all other reagents were obtained from Sigma Chemical.

### Fast scan cyclic voltammetry (FSCV)

Male Sprague Dawley rats, 21–26 d old, or *Th::Cre* transgenic rats (Witten et al., 2011), 46–51 d old at the time of virus injection, were used in these studies. Th::Cre rats were injected with the Cre-dependent ChR2-expressing virus (AAV2-Ef1a-DIO-hChR2(H134R)-mCherry, titer 5.1 × 1012 viral particles/mL, UPenn viral core) bilaterally into the VTA 500 nL per side (AP, −5.3 mm; ML, ± 0.4 mm; DV, −8.2 mm from bregma). Five weeks later, coronal slices (400 µm) containing the NAc were prepared for voltammetry measurements. The use of Cre dependent ChR2 expression allowed selective optical control of VTA dopamine terminals in the NAc.

Extracellular dopamine release was achieved using either electrical (in wild-type Sprague Dawley rats) or 470-nm light (in *Th::Cre* rats) stimulation. Stimulation parameters were the same for both electrical and optical stimulation (10 Hz, two pulses, 4 ms). Electrochemical recordings were made using carbon fiber electrodes fabricated from T-650 carbon fiber (7 µm diameter, gift from Leslie Sombers, NCSU) that was aspirated into a borosilicate glass capillary (0.6 × 0.4 mm or 1.0 × 0.5 mm in diameter, King Precision Glass Inc.) and pulled using a PE-22 puller (Narishige). Carbon fiber electrodes were positioned 80 µm into the tissue either between the bipolar tips of the stimulating electrode or directly in front of an optical fiber connected to an LED emitting 470 nm light (7–10 mW). The potential of the carbon fiber electrode was held at −0.4 V relative to the Ag/AgCl reference electrode. A triangle wave form was applied to the carbon fiber driving the potential from −0.4 to +1.3 V and back to −0.4 V at a rate of 400 V/s, at 60 Hz for conditioning and 10 Hz for data collection. Data were collected with a WaveNeuro FSCV potentiostat (Pine Research) using HDCV acquisition software package (freely available through UNC Department of Chemistry). HDCV Acquisition Software was used to output the electrochemical waveform and for signal processing (background subtraction, signal averaging, and digital filtering; four-pole Bessel filter, 2.5 kHz). Dopamine release was stimulated at 2 min intervals for electrical stimulation and 3 min intervals for optical stimulation. The difference in stimulation intervals was to decrease rundown of the dopamine release signal that can be particularly strong in optical experiments as reported in (Bass et al., 2013; O'Neill et al., 2017). Mean background currents from 1 s of data before stimulation were removed by subtraction of cyclic voltammograms for each trial.

### Data analysis

For electrophysiology, effects of N/OFQ were statistically evaluated in each neuron by binning data into 30 s data points and comparing the last eight binned predrug points to the last eight binned points during drug application using Student’s unpaired *t* test. To evaluate the output of this analysis approach, we performed a subsequent sliding window analysis on this classified data from TH(+) neurons that were tested with 10 nm N/OFQ (Extended Data Fig. 1-1). The results of this analysis are consistent with this classification scheme identifying drug responses and a lack of contamination by drift in individual recordings. The summary effect sizes reported here are the differences between the mean of this baseline 4 min window and the mean of the *I*_holding_ during the last 4 min of drug application. For within cell comparisons of N/OFQ responses, responses were compared with a Student’s paired *t* test; *p *<* *0.05 was required for significance in all analyses. Differences between neuron populations were tested using two-tailed permutation analyses unless otherwise indicated. Violin plots were constructed by calculating the kernel density estimate, made using a Scott estimator for the kernel bandwidth estimation. The kernel size was determined by multiplying the Scott bandwidth factor by the SD of the data within each bin. Each individual violin plot was normalized to have an equal area under the curve. Time course figures are averages of the binned current traces for all cells time locked to the start of drug application. EC_50_ was estimated by fitting the concentration response data with the Hill equation. Results are presented as mean and SEM. Custom code created for analyses here are publicly available at https://osf.io/c8gu7/?view_only=24595243ef6d44d5974442b23dda0b1d.

## Results

### N/OFQ effects on holding current in VTA dopamine and non-dopamine neurons

**Figure 1.**
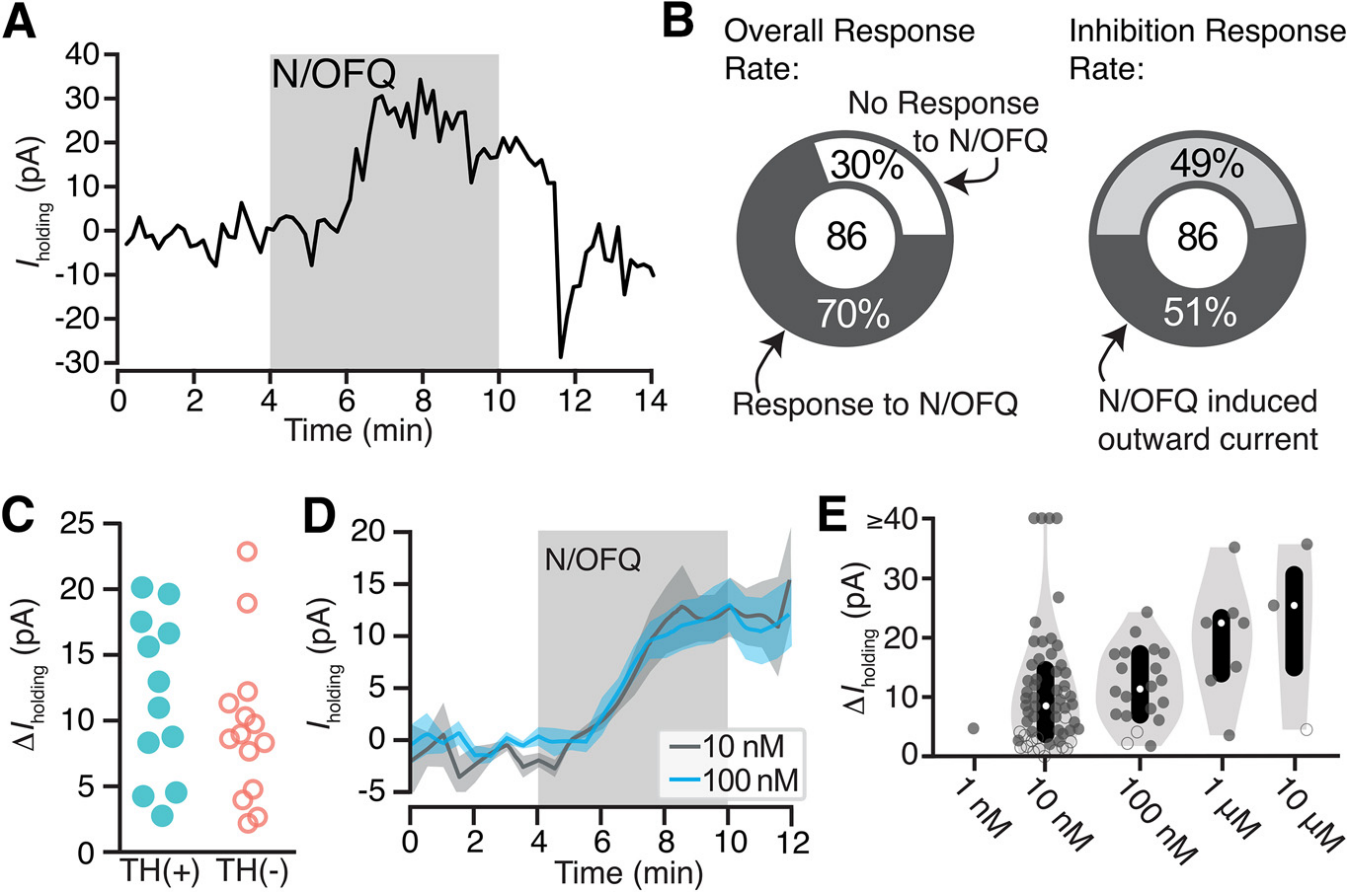
N/OFQ-induced outward currents in a subset of VTA neurons. ***A***, Example voltage-clamp recording (V_clamp_ = −60 mV) of a VTA neuron that responded to N/OFQ with an outward current. ***B***, Across recordings in neurons from control rats, the majority of VTA neurons responded to 10 nm N/OFQ application (60 out of 86 neurons responded). Forty-four out of 60 responses were outward currents. ***C***, A subset of recorded neurons were recovered following whole-cell recording and immunocytochemically identified for TH content, a marker for dopamine neurons. Outward currents of similar magnitudes were observed in TH(+) and TH(–) neurons. ***D***, The mean ± SEM time courses and maximal effects of bath application of 10 and 100 nm N/OFQ were similar. ***E***, Concentration response relationship for VTA neurons showing a positive change, both significant (solid circle) and not significant (open circle), in holding current with N/OFQ application (gray markers include all neurons with a change >0 pA; median shown in white dots; black bars show 25th and 75th percentiles; 1 nm: *n* = 1/6; 10 nm: *n* = 55/86; 100 nm: *n* = 20/25; 1 μm: *n* = 7/8; 10 μm: *n* = 3/4).

To test the postsynaptic responses of VTA neurons to N/OFQ, we made *ex vivo* whole-cell voltage-clamp recordings (V_m_ = −60 mV). N/OFQ application changed the holding current in 70% (60/86) of neurons tested in the VTA (10 nm; 86 neurons from 59 rats; Fig. 1*A*,*B*). The majority of responses were relatively small outward currents (73% of responsive neurons, 44/60; 51% of all neurons tested, 44/86; mean response magnitude = 15 ± 2 pA; Fig. 1*D*; examples of small responses provided in Extended Data Fig. 1-2*A-C*). In many cases, the holding current returned to baseline during N/OFQ washout, as in Figure 1*A*; however, in some cases, we observed only partial recovery. Using *post hoc* immunocytochemistry, we analyzed TH content in each histologically recovered neuron and found that N/OFQ inhibited both confirmed dopamine and non-dopamine neurons in similar proportions [of 44 inhibited neurons from 38 rats, 26 neurons from 23 rats were identified: TH(+): 12/26; TH(–): 14/26]. The magnitudes of responses were also similar between confirmed dopamine and non-dopamine neurons [TH(+): 12 ± 2 pA (*n* = 12); TH(–): 9 ± 2 pA (*n* = 14); *p *=* *0.3 two-tailed permutation test; Fig. 1*C*]. The EC_50_ for these outward currents is in the nM range (8 ± 6 nm; Fig. 1*E*).

10.1523/ENEURO.0376-19.2020.f1-1 Extended Data Figure 1-1 To evaluate our within cell statistical comparisons to identify “responsive” versus “non-responsive” neurons, in particular to test the possibility that drift might contribute to some of our identified drug effects, we conducted a sliding window analysis on a subset of our drug responses (all TH-positive neurons tested with bath application of 10 nm N/OFQ). Further, any increase in statistically significant sliding windows during drug washout compared to the static baseline would suggest underlying *I*_holding_ drift. We compared all 4-min windows from predrug application through drug washout to a fixed “baseline” window (the 4 min preceding the onset of the drug). To create the windows, *I*_holding_ of each recording was binned into 30-sec intervals and assigned a bin number (1, 2, 3 Ö n). The baseline eight-bin (4-min) window was compared with the target 4-min window by way of a Student’s unpaired *t* test. The *p* value and significance of the comparison were then corrected using the Bonferroni method for multiple comparisons. The alignment of the sliding window was then increased by a single bin and the comparison repeated, resulting in an array that represents all significant 4-min intervals for each drug effect. The resulting arrays were plotted as a histogram representing, at the initial bin time of the sliding window, the proportion of recordings in which this calculation was significantly different from the fixed baseline target window (***A***). In the neurons previously classified as responsive by a single baseline compared to “drug” window comparison, the rising left edge of the histogram begins to plateau around the 4th minute of drug application, consistent with the plateau of the mean effects across all cells reported in Figure 1*D*. Further, consistent with washout reversal of N/OFQ effects in most but not all neurons, the proportion of significant bins falls off as soon as N/OFQ application was terminated. That both the rise and fall of the frequencies of significant windows are time locked to the drug application suggests the response classification scheme is reliable. In neurons previously classified as non-responsive only one neuron had any significant windows, with three sliding window locations where this analysis yielded *p *<* *0.05, suggesting that there was not systematic drift in these non-responsive neurons. In addition, a scatter plot (***B***) indicates the maximum number of consecutive significant sliding windows for each cell analyzed, because a well-behaved change in *I*_holding_ in response to the drug application should be detected in consecutive sliding windows. This graph shows that 8/12 neurons that were classified as responsive have more consecutive sliding windows different from baseline than the maximum found in non-responsive neurons. This analysis was conducted using a custom script created in Python (available at https://osf.io/c8gu7/?view_only=24595243ef6d44d5974442b23dda0b1d). Download Figure 1-1, EPS file.

**Figure 2:**
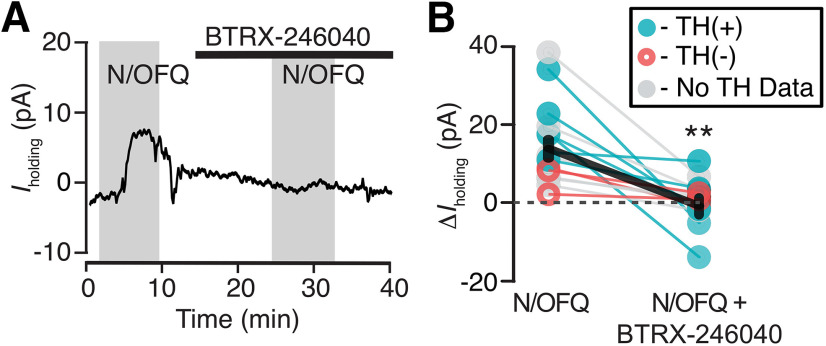
BTRX-246040 consistently blocks N/OFQ-induced currents. ***A***, Example recording of a N/OFQ (10 nm) responsive neuron where the selective NOP antagonist BTRX-246040 (100 nm) blocked the response to a subsequent N/OFQ application. ***B***, BTRX-246040 blocked N/OFQ responses across VTA neurons, including both TH(+) and TH(–) neurons (*n* = 6 and 3, respectively; *n* = 6 no TH data; mean ± SEM in black); **p <* *0.01.

**Figure 3:**
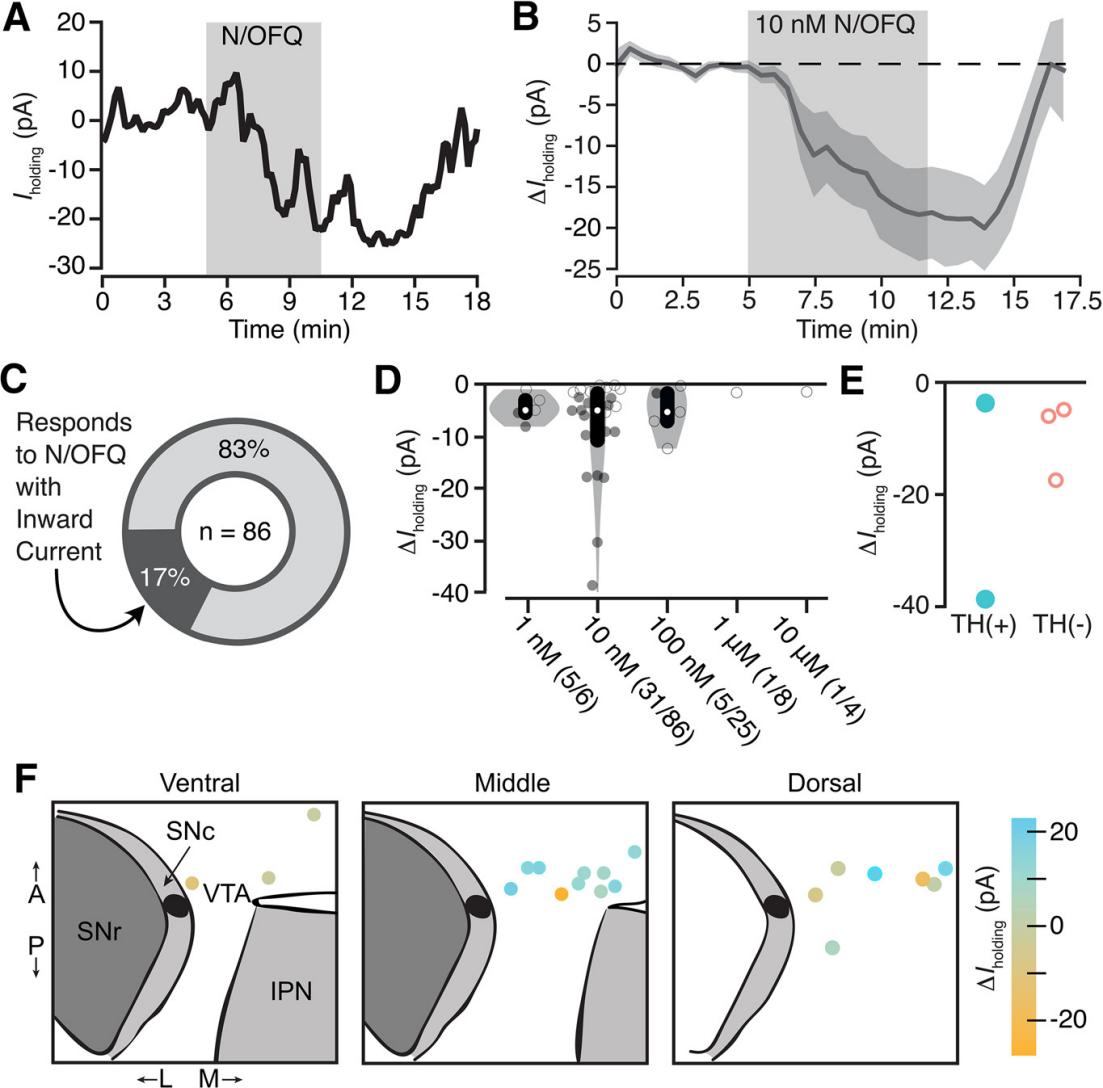
Low-dose N/OFQ-induced small inward currents in a subset of VTA neurons. ***A***, Example voltage-clamp recording (V_clamp_ = −60 mV) of a VTA neuron that responded to N/OFQ with an inward current. ***B***, The mean ± SEM time course across neurons with inward currents shows the onset of this response is time locked to the initiation of drug application (*n* = 15). ***C***, Across all VTA neurons from control rats that were tested for 10 nm N/OFQ responses, 17% responded with a significant inward current. ***D***, Concentration response data for each neuron showing a negative change, both significant (filled circle) and not significant (open circle), in holding current with N/OFQ application (gray markers include all neurons with a change <0 pA; median shown in white dots; black bars show 25th and 75th percentiles). Significant inward currents were observed at 10 nm, while higher concentrations only generated outward currents (Fig. 1*E*). ***E***, Inward currents were observed in both immunocytochemically identified TH(+) and TH(–) neurons. ***F***, Locations of VTA recordings show that neurons that responded to N/OFQ with inward and outward currents were intermixed.

10.1523/ENEURO.0376-19.2020.f1-2 Extended Data Figure 1-2 These graphs show the whole-cell voltage-clamp recordings of the three smallest changes in holding current that were categorized as responses to 10 nm N/OFQ by statistical analysis for Figures 1*B*, 3*C*, outward, left; inward, right. Grey boxes indicate drug application. Blue boxes indicate baseline measurement interval, green boxes indicate drug effect measurement interval. Download Figure 1-2, EPS file.

To confirm responses were due to activation of the NOP, we tested whether these inhibitions were blocked by the selective NOP antagonist BTRX-246040. In neurons responding to N/OFQ with an outward current, BTRX-246040 (100 nm) was applied for 10 min and then N/OFQ was applied a second time in the presence of the antagonist. BTRX-246040 consistently blocked N/OFQ-induced outward currents (baseline 10 nm N/OFQ response: 14 ± 3 pA; N/OFQ response in BTRX-246040: −1 ± 2 pA; *n* = 15; 14 rats; paired *t* test: *p *=* *0.0005; Fig. 2).

We also observed a subpopulation of neurons that responded to N/OFQ application with a small inward current, consistent with an excitatory effect (10 nm mean response = −16 ± 6 pA; Fig. 3*A*,*B*). Inward currents were observed in ∼25% (15/60) of the neurons that were responsive to N/OFQ (10 nm) and 17% of all 10 nm-tested VTA neurons (15/86; 15 neurons from 14 rats; Fig. 3*C*,*D*; examples of small responses provided in Extended Data Fig. 1-2*D-F*). Among five neurons responding to N/OFQ with an inward current and immunocytochemically identified, 40% (2/5) were TH(+) and 60% (3/5) were TH(–) (two-tailed permutation test: *p *=* *0.6; Fig. 3*E*). These N/OFQ evoked excitatory responses were only observed at low concentrations (≤100 nm; Fig. 3*D*); at higher concentrations, only outward currents were observed (Figs. 1*E*, 3*D*). The neurons showing this excitatory response to N/OFQ were topographically intermixed with VTA neurons that responded to N/OFQ with an outward current (Fig. 3*F*).

### Concentration-dependent desensitization of NOPs

**Figure 4.**
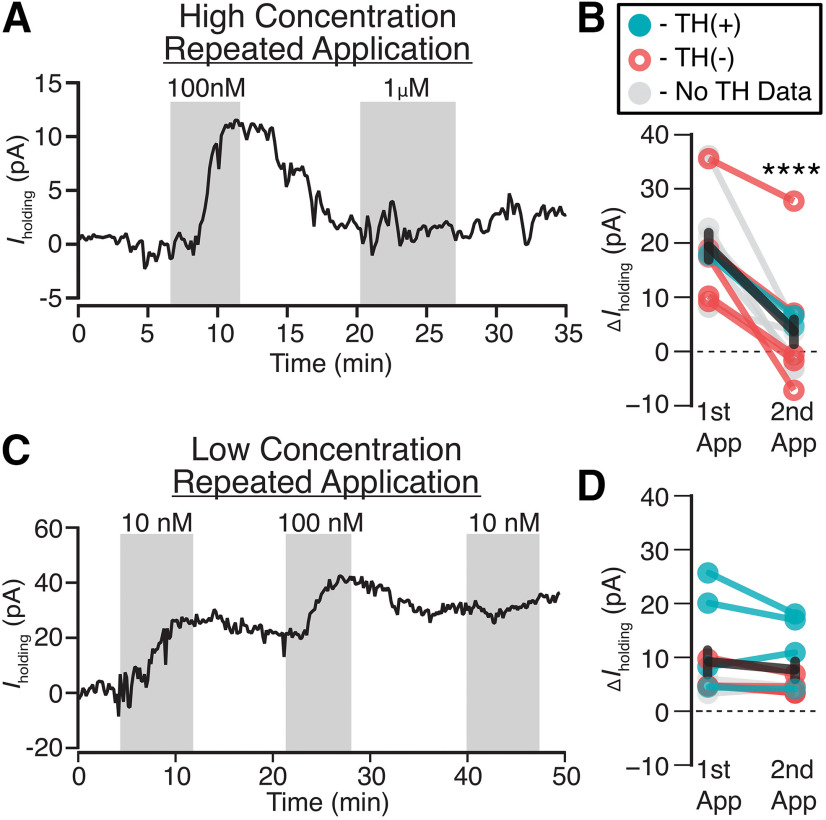
Moderate concentrations of N/OFQ cause functional desensitization in VTA neurons. ***A***, Example voltage-clamp recording (V_clamp_ = −60 mV) where 100 nm is sufficient to prevent a subsequent response to 1 µm application of N/OFQ. ***B***, A summary across VTA neurons where the first N/OFQ application was ≥100 nm, the response to the second application was consistently smaller (****p =* *0.00003), in both TH(+) and TH(–) neurons (*n* = 2 and 5, respectively; *n* = 5 no TH data). ***C***, Example voltage-clamp recording (V_clamp_ = −60 mV) showing that 10 nm N/OFQ does not impair responses to subsequent N/OFQ application. In the same cell, 100 nm did prevent additional responding. ***D***, Summary across VTA neurons shows similar magnitudes of responses to the second application of N/OFQ when the first application was 10 nm [*p *=* *0.13; TH(+) *n* = 4, TH(–) *n* = 2, TH no data *n* = 2].

Given the inconsistencies in the reports of behavioral effects of NOP agonists and antagonists, we tested whether N/OFQ causes rapid NOP desensitization at moderate doses. We observed a concentration-dependent diminished response to a second application of N/OFQ when the first application of N/OFQ was ≥ 100 nm (*n* = 12 neurons from 12 rats; paired *t* test *p *=* *0.00003; Fig. 4*A*,*B*). This is consistent with NOP desensitization, and observed in both TH(+) and TH(–) neurons (Fig. 4*B*). In contrast, following administration of 10 nm N/OFQ, no significant difference in response was observed between the second and first applications (*n* = 10 neurons from 8 rats; paired *t* test *p *=* *0.13; Fig. 4*C*,*D*). Therefore, desensitization occurs at moderate N/OFQ concentrations in the VTA.

### N/OFQ inhibits VTA neurons and SNc neurons via different cellular mechanisms

**Figure 5.**
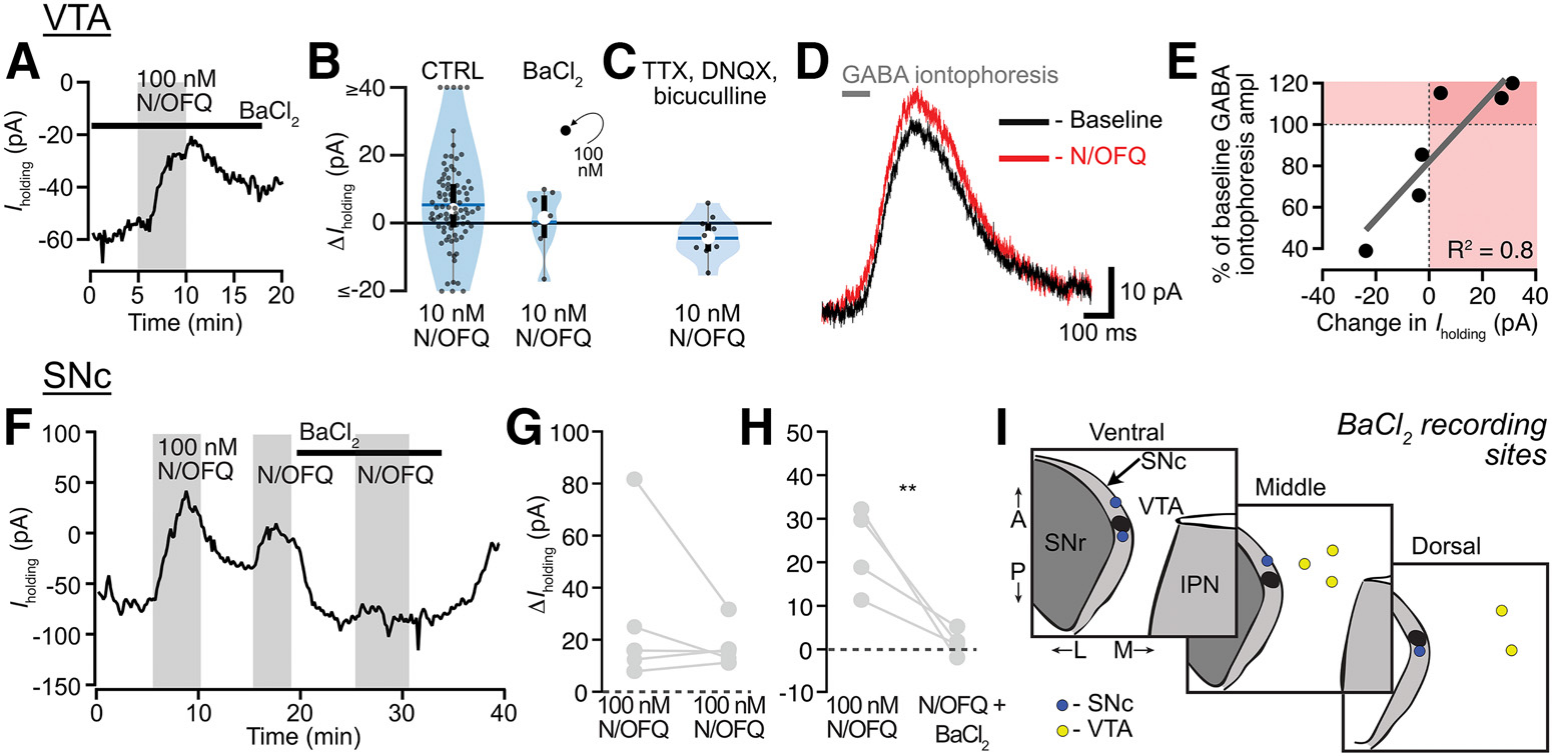
GABA_A_Rs, rather than GIRKs, mediate N/OFQ effects in VTA neurons. ***A***, Example recording showing that the K^+^ channel blocker BaCl_2_ (100 μm) did not prevent a N/OFQ-induced outward current in a VTA neuron. ***B***, Blue violin plots representing the distributions of responses of VTA neurons to 10 nm N/OFQ (blue horizontal line = mean; white circle = median; black rectangle = 25th and 75th percentiles). Gray circles show individual responses (single 100 nm experiment in black). There was no difference detected between the distribution of control observations in p22–p36 from adult animals in the presence of BaCl_2_ have a similar distribution (one tailed permutation analysis of the means, *p *=* *0.2). ***C***, Recordings in 500 nm TTX, 10 μm DNQX, and 10 μm bicuculline to block synaptic activity, AMPARs, and GABA_A_Rs, respectively, showed an almost complete elimination of outward currents in VTA neurons in response to N/OFQ (two-tailed permutation analysis of the means, *p *=* *0.16; one tailed permutation analysis of the SDs, *p *=* *0.03). ***D***, Example recording of GABA_A_R-mediated iontophoretic responses to GABA (in 30 μm CGP35348 to block GABA_B_Rs) showing an augmentation of response amplitude in response to 100 nm N/OFQ. ***E***, Summary of the N/OFQ (100 nm)-induced change in iontophoretic response versus change in *I*_holding_, showing both inward and outward N/OFQ-induced currents are highly correlated with N/OFQ-induced changes in iontophoresis amplitude (*t* = 3.904; df = 4; *p *=* *0.02). ***F***, Example recording in a SNc neuron showing repeated responses to high-concentration (100 nm) N/OFQ, and blockade of the N/OFQ response by BaCl_2_. ***G***, Summary data from SNc neurons showing minimal desensitization in control experiments with repeated within cell N/OFQ applications at high concentration. ***H***, Summary data from SNc neurons show that BaCl_2_ prevents a second response to N/OFQ, indicating that in the SNc, N/OFQ outward currents are mediated by K^+^ channels; **p <* *0.01. ***I***, Recording locations for VTA and SNc recordings where N/OFQ was tested in the presence of BaCl_2_.

We investigated the mechanism underlying the outward currents produced by N/OFQ in VTA neurons. These experiments were completed in adult animals to ensure we measured the mature mechanisms of N/OFQ actions. The most common mechanism by which Gi/o coupled receptors, including the NOP, generate somatodendritic electrophysiological inhibition is by activation of GIRKs. First, we tested whether the K^+^ channel blocker BaCl_2_ (100 µm) prevented N/OFQ-induced outward currents. Surprisingly, BaCl_2_ did not prevent the outward currents induced by N/OFQ at either 100 nm (Fig. 5*A*) or 10 nm [Fig. 5*B*; one tailed permutation analysis comparing all 10 nm N/OFQ VTA observations from p22–p36 animals in Fig. 1 (*n* = 86) to 10 nm N/OFQ observations in the presence of 100 µm BaCl_2_ (*n* = 7), *p *=* *0.2; each recording with a outward current response >1.5 pA is shown in Extended Data Fig. 5–1*A-C*]. We next tested whether a cocktail of synaptic blockers including the Na^+^ channel blocker tetrotodoxin (TTX; 500 nm), the AMPA receptor (AMPAR) blocker 6,7-dinitroquinoxaline-2,3(1H,4H)-dione (DNQX; 10 μm), and the GABA_A_R antagonist bicuculline (10 μm) would alter N/OFQ responses. Interestingly, while this cocktail did not significantly change the mean of VTA neuron N/OFQ responses [Fig. 5*C*; the one recording with a outward current response >1.5 pA is shown in Extended Data Fig. 5-1*D*; two-tailed permutation analysis comparing the means of all 10 nm N/OFQ VTA observations from p22–p36 animals in Fig. 1 (*n* = 86) to 10 nm N/OFQ observations in the synaptic blocker cocktail (*n* = 9), *p *=* *0.16)], only one out of nine neurons responded to 10 nm N/OFQ with an outward current under these conditions (Extended Data Fig. 5-1*D*), raising the possibility of a difference in the proportion of neurons with this type of response in the inhibitor cocktail. The SD of the distribution of N/OFQ responses in the presence of the inhibitor cocktail was significantly reduced, also consistent with the possibility this treatment diminished N/OFQ responses (one tailed permutation analysis comparing the SDs of all 10 nm N/OFQ VTA observations from p22–p36 animals in Fig. 1; SD = 20.36 pA, variance = 414.53 pA, *n* = 86) to 10 nm N/OFQ observations in the synaptic blocker cocktail (SD = 6.27 pA, variance = 39.27 pA, *n* = 9), *p *=* *0.03; 10 nm N/OFQ observations in BaCl_2_ (SD = 9.34 pA, variance = 87.27 pA, *n* = 9), *p *=* *0.15]. Since the cocktail of synaptic blockers did not yield a significant change in the mean of the responses, this was most consistent with both outward and inward current responses being diminished. Focusing on the outward currents, if these blockers decreased the outward current responses to N/OFQ, the simplest possible mechanisms are via an inhibition of AMPAR signaling, via an increase in GABA_A_R signaling, or via a non-GIRK-dependent effect of a substance released by action potential activity in the slice. We previously found that in stressed animals, DOP activation in the VTA postsynaptically increases GABA_A_R signaling in VTA neurons (Margolis et al., 2011), and there is evidence for tonic GABA_A_R currents in VTA neurons (Darnieder et al., 2019). On the other hand, spontaneous glutamate release in the VTA seems insufficient to support generating an apparent outward current when glutamate release is inhibited (Koga and Momiyama, 2000; Margolis et al., 2005; Xiao et al., 2009). Therefore, we tested whether N/OFQ affects GABA_A_R signaling in the VTA and whether this might account for N/OFQ-induced changes in holding current. We iontophoretically applied GABA in the presence of GABA_B_R blockade (CGP35348, 30 μm) to measure GABA_A_R responses and to bypass any potential presynaptic terminal effects. We not only found that 100 nm N/OFQ increased the amplitude of GABA_A_R responses (Fig. 5*D*,*E*), the effect on iontophoresed GABA currents was proportional to the change in holding current induced by N/OFQ (Fig. 5*E*), across both inward and outward currents induced by N/OFQ, making it likely that GABA_A_R signaling underlies both inward and outward currents induced by N/OFQ application to VTA neurons.

10.1523/ENEURO.0376-19.2020.f5-1 Extended Data Figure 5-1 These graphs show each of the whole-cell voltage-clamp recordings represented in the summary dot plots in Figure 5*B*,*C* where the 10 nm N/OFQ-induced change in holding current >1.5 pA. In control experiments, 55/86 neurons (64%) responded to 10 nm N/OFQ with a change in holding current >1.5 pA. In the case of BaCl_2_ experiments, 3/7 (43%) of the tested neurons responded with a change in holding current >1.5 pA (***A–C***). In one of these neurons, we tested the N/OFQ response twice, and the neuron responded with an outward current both times (top example). For experiments in which we tested for 10 nm N/OFQ responses in the presence of TTX, DNQX, and bicuculline, only 1/9 neurons (11%) responded with a change in holding current >1.5 pA (***D***). In this example, the change in holding current induced by the cocktail of blockers is apparent during minutes 0–3. Download Figure 5-1, EPS file.

That N/OFQ-induced outward currents are because of augmentations of GABA_A_R-mediated current rather than activation of a K^+^ current was particularly surprising because it was previously reported that N/OFQ activates a K^+^ channel in VTA neurons (Zheng et al., 2002). Zheng and colleagues also reported larger average outward currents compared with our dataset and did not observe desensitization with repeated applications of 300 nm N/OFQ, inconsistent with our findings here. As a positive control to test that 100 μm BaCl_2_ was sufficient to block K^+^-mediated effects in our preparation, and in an attempt to resolve these discrepancies, we completed additional recordings in the SNc, just lateral to the VTA (Fig. 5*I*). First, we tested whether repeated application of 100 nm N/OFQ to SNc neurons resulted in less desensitization than we observed in VTA neurons. In fact, the response to the second 100 nm N/OFQ application was not statistically different from the response to the first application in SNc neurons, in contrast to VTA neurons (Fig. 5*F*,*G*; two-tailed paired *t* test, *p *=* *0.5, *n* = 5). Therefore, we used a within cell design to compare the N/OFQ response in control aCSF and in 100 µm BaCl_2_. Blocking K^+^ channels significantly reduced the magnitude of N/OFQ responses in SNc neurons (Fig. 5*H*; one-tailed paired *t* test, *p *=* *0.003, *n* = 5). Together, these observations indicate that BaCl_2_ was fully capable of blocking GIRK-mediated N/OFQ effects in our recording conditions and suggest that the differences between our observations and those previously reported may be related to recording location (Fig. 5*I*).

### N/OFQ effects on VTA neurons vary with projection target

**Figure 6.**
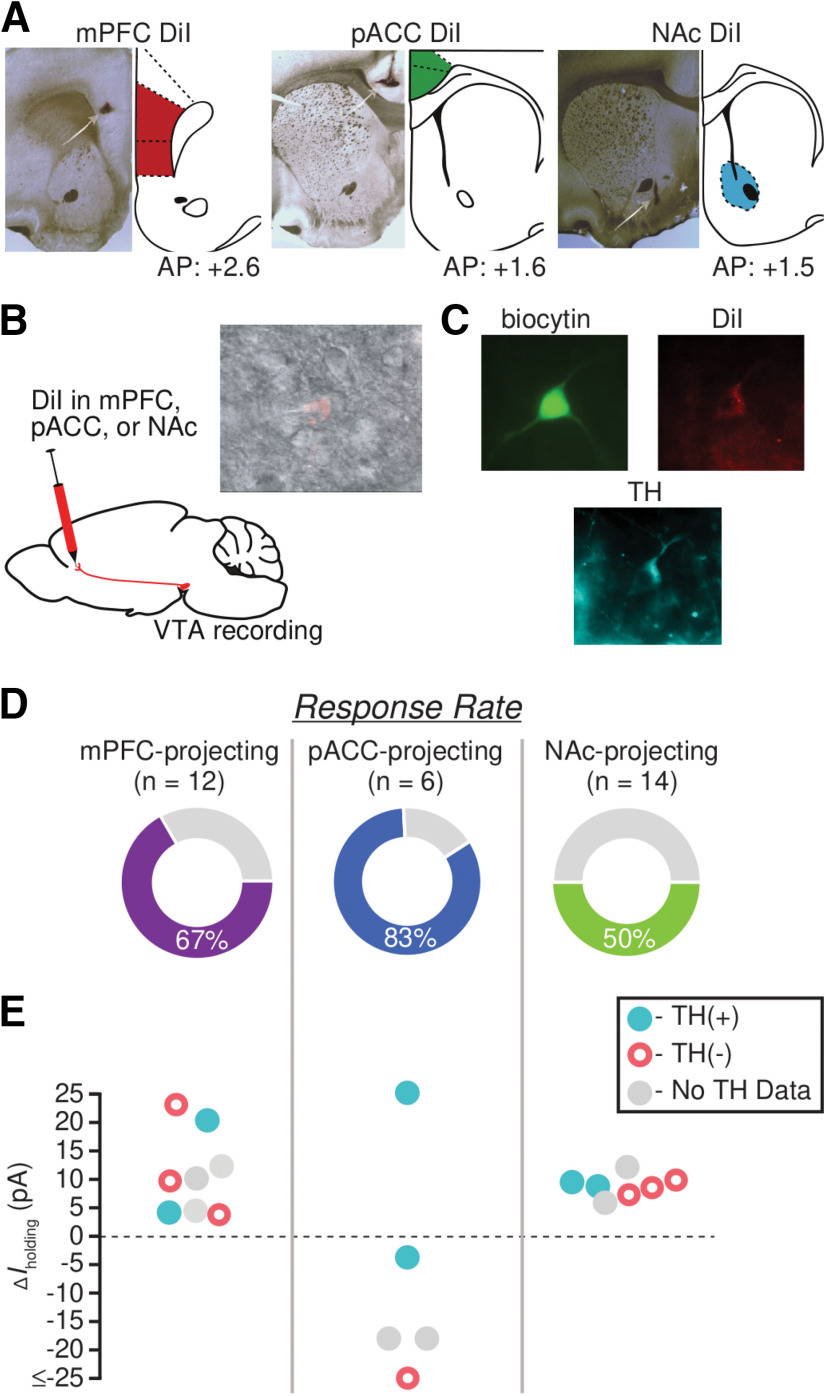
N/OFQ effects in VTA neurons vary with projection target. ***A***, For each retrograde tracer injection site, example histology photograph showing DiI localization (left) and mirrored, modified rat brain atlas schematic (right; Paxinos and Watson, 1997). ***B***, Cartoon showing the experimental approach: 7 d before recording, the retrograde tracer DiI was stereotaxically injected into mPFC, pACC, or medial NAc. DiI neurons were identified during whole-cell recordings (inset). ***C***, Example image of a neuron filled with biocytin during recording (green), the retrograde tracer (red), and was immunocytochemically identified as TH(+) (turquoise). ***D***, The overall percentage of neurons that responded to N/OFQ was greatest among pACC-projecting neurons and lowest among NAc-projecting neurons. ***E***, Graph of magnitudes of significant N/OFQ responses, showing that only pACC-projecting neurons respond to N/OFQ with an inward current.

As described above, we observed heterogeneity in responses of VTA neurons to N/OFQ. Given that other pharmacological responses of VTA neurons, including to KOP activation (Ford et al., 2006; Margolis et al., 2006), vary with projection target, we investigated whether the N/OFQ responses would be more consistent within subpopulations of VTA neurons that share a projection target. Accordingly, we recorded N/OFQ (10 nm) responses in VTA neurons that were retrogradely labeled by tracer injections into mPFC, pACC, or medial NAc (Fig. 6*A*,*B*). Recordings were conducted with the investigator blinded to the injection site.

The majority of mPFC-projecting VTA neurons, 67% (8/12), were significantly inhibited by N/OFQ, responding with an outward current (11 ± 3 pA; eight responsive neurons from six rats; Fig. 6*D*,*E*). No N/OFQ-induced inward currents were observed in mPFC-projecting neurons. Five mPFC-projecting neurons were recovered and processed for TH immunoreactivity (Fig. 6*C*,*E*); two were TH(+), and three were TH(–); all of these responded to N/OFQ with an outward current (Fig. 6*E*).

VTA projections to different cortical targets, including the pACC, arise from largely separate VTA neurons (Chandler et al., 2013). The pACC-projecting neurons are concentrated in different parts of the VTA, and fewer of them are dopaminergic compared with the projection to mPFC (Breton et al., 2019). Interestingly, 67% of the VTA neurons comprising this projection responded to N/OFQ with an inward current (4/6 inward current, −24 ± 12 pA, 1/6 outward current, from four rats; Fig. 6*D*,*E*). These N/OFQ excited, pACC-projecting VTA neurons included both TH(+) and TH(–) cells (Fig. 6*E*).

Half of NAc-projecting VTA neurons (7/14) responded to N/OFQ with outward currents (9 ± 1 pA, seven responsive neurons from seven rats; Fig. 6*D*,*E*). No inward currents were observed in this projection. Of the seven NAc-projecting neurons that responded to N/OFQ, two were confirmed TH(+) and three were TH(–) (Fig. 6*E*). Together, these data indicate that similar N/OFQ inhibitory effects occur in VTA neurons that project to mPFC and NAc, but these effects are opposed to those on VTA projections to pACC, many of which responded to N/OFQ with an inward current.

### N/OFQ has minimal effect on terminal dopamine release in the NAc

**Figure 7.**
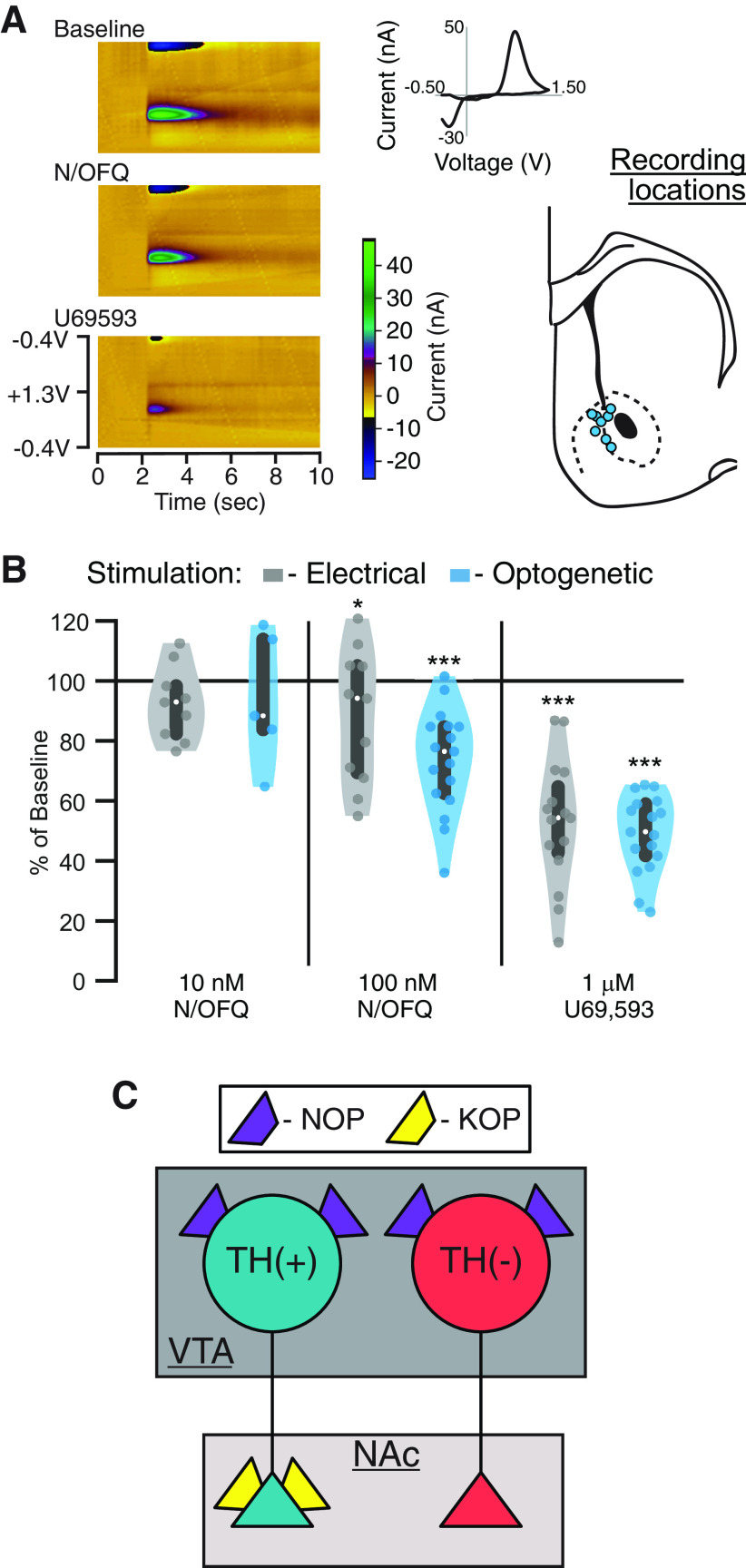
N/OFQ minimally inhibits dopamine release at NAc terminals. We used FSCV in acute, coronal slices containing the NAc to test for N/OFQ effects on terminal release of dopamine. Dopamine release was evoked in slices from control rats with bipolar electrodes locally in the NAc. Recordings were made on the NAc shell-core border. Alternatively, to limit stimulation to dopamine axons, *Th::Cre* rats were injected with (AAV2-Ef1a-DIO-hChR2(H134R)-mCherry) in the VTA at least four weeks before recordings, and 470-nm light pulses were used to stimulate dopamine release. ***A***, Example color plots of FSCV measurement of electrically evoked dopamine release. Inset, top, Background subtracted cyclic voltammogram at peak of putative dopamine release. Inset, bottom, Locations of FSCV recordings in schematic of coronal section of rat brain AP: +1.5 mm (Paxinos and Watson, 1997). ***B***, Serial application of 10 nm then 100 nm N/OFQ was applied to the slice; 10 nm did not induce a significant change in either electrically or light evoked dopamine release; 100 nm had a small but significant inhibitory effect on electrically (*p* = 0.04) or light (*p *<* *0.001) evoked dopamine release in the NAc (linear mixed effects model). Following N/OFQ measures, without washout, we added the KOP agonist U69,593 (1 μm), which inhibited evoked dopamine release. White dots represent median values and gray bars represent 25th and 75th percentiles. ***C***, Summary diagram shows the contrast between NOP and KOP function in NAc-projecting VTA dopamine neurons. While NOP activation inhibits the somatodendritic compartment only, KOP-induced inhibition is limited to dopaminergic axon terminals in these neurons. Further, NOP activation inhibits NAc-projecting non-dopaminergic VTA cell bodies, which are insensitive to KOP activation.

ICV or intra-VTA N/OFQ decreases dopamine levels in the NAc (Murphy et al., 1996; Murphy and Maidment, 1999). Consistent with this result, we found that N/OFQ directly inhibits a subset of the NAc-projecting VTA dopamine somata. N/OFQ may also inhibit dopamine release in the NAc at the terminals; to test whether NOPs on dopamine terminals in the NAc also contribute to an N/OFQ-induced decrease in NAc dopamine levels, we used FSCV to detect changes in stimulated dopamine release in NAc slices (Fig. 7*A*). In tissue from control SD rats (nine rats), we stimulated dopamine release with a bipolar electrode. In a second set of animals, to limit stimulation to dopaminergic axons, we expressed ChR2 in *Th::Cre* rats and stimulated with 470-nm light pulses (nine rats). In these preparations, repeated electrical, and especially optical, stimulation can cause rundown in evoked dopamine release over time (Bass et al., 2013; O'Neill et al., 2017). To minimize this rundown as much as possible, we increased the intervals between light stimulations to 3 min. Where recordings were stable, effects of 10 nm N/OFQ, 100 nm N/OFQ, and 1 µm U69,593 were sequentially tested. At 10 nm N/OFQ, approximately the EC_50_ of the outward currents recorded at VTA somata, there was no change in the peak FSCV response to either electrically or light evoked dopamine release (electrically evoked dopamine release: 93 ± 4% of baseline, *n* = 9 slices from 9 rats: linear mixed effects model, *z* = −1.3, *p *=* *0.2; optically evoked dopamine release: 94 ± 10% of baseline, *n* = 5 from 5 rats: linear mixed effects model, *z* = −0.5, *p *=* *0.6; Fig. 7*A*,*B*). We detected a small but significant decrease in evoked dopamine release in response to 100 nm N/OFQ (electrically evoked dopamine release: 88 ± 7% of baseline, *n* = 11 from 9 rats: linear mixed effects model, *z* = −2.1, *p *=* *0.04; optically evoked dopamine release: 74 ± 4% of baseline, *n* = 17 from 9 rats: linear mixed effects model, *z* = −7.2, *p *<* *0.001; Fig. 7*B*). Consistent with previous studies (Bass et al., 2013; O'Neill et al., 2017), it is possible that this small decrease was driven, at least in part, by rundown of ChR2-driven dopamine release. As a positive control, we applied the selective KOP agonist U69,593 (1 µm), previously shown to inhibit dopamine release in the NAc (Di Chiara and Imperato, 1988a,b; Werling et al., 1988; Spanagel et al., 1992; Ebner et al., 2010; Karkhanis et al., 2016), at the end of each experiment, on top of N/OFQ since these drug responses were minimal. U69,593 caused a substantial decrease in stimulated dopamine release (electrical: 53 ± 5% of baseline (in N/OFQ), *n* = 15: linear mixed effects model, *z* = −8.9, *p *<* *0.001; optical: 49 ± 3% of baseline (in N/OFQ), *n* = 17: linear mixed effects model, *z* = −13.9, *p *<* *0.001; Fig. 7*B*). Therefore, the direct NOP modulation of this dopaminergic circuit occurs at a lower concentration and may be stronger in the somadendritic region where the terminals in the NAc are relatively insensitive to NOP activation. These results contrast with the KOP control of these neurons, which strongly inhibits release at the NAc dopamine terminals but does not directly hyperpolarize the cell bodies of these neurons (Margolis et al., 2006; Fig. 7*C*).

## Discussion

The results presented here demonstrate that N/OFQ affects both dopaminergic and non-dopaminergic VTA neurons, through activation of the NOP, and in the majority of neurons causes inhibitory outward currents. N/OFQ effects in these neurons were blocked by the NOP-selective antagonist BTRX-246040, confirming its action at NOP. Importantly, neuronal responses to N/OFQ in VTA neurons desensitized at concentrations ≥100 nm. In addition to providing a basic characterization of the range of postsynaptic N/OFQ responses in VTA neurons, we demonstrated differential responding of subsets of VTA neurons to NOP activation related to projection target: mPFC-projecting and NAc-projecting VTA neurons responded to N/OFQ with outward currents (inhibitory), while most pACC-projecting VTA neurons responded with inward currents (excitatory). Within the dopaminergic projection to the NAc, although N/OFQ caused outward currents at the somatodendritic region of these neurons, release at the terminals was not inhibited by NOP activation. Together, these data show that N/OFQ effects in VTA neurons differ depending on their projection target and that at higher concentrations of N/OFQ only inhibitions are observed, followed by desensitization of NOP function.

Unexpectedly, a small population of neurons in the VTA, both TH(+) and TH(–), responded to low concentrations of N/OFQ with an inward current, consistent with excitation. This finding presents a novel mechanism by which N/OFQ could selectively activate specific VTA circuits, while inhibiting the majority of VTA outputs. Inward currents were observed in most VTA neurons projecting to the pACC, but not those projecting to the NAc or mPFC, consistent with this circuit-selection proposition. The fact that this effect was only observed at low concentrations indicates that very robust N/OFQ release into the VTA, on the other hand, would likely have a broad inhibitory effect on the vast majority of VTA neurons, regardless of circuit. Although NOPs are generally thought to couple to Gi/o and inhibit neural activity, some exceptions to this coupling have been reported for the related opioid receptors. Activation of postsynaptic MOP or DOP results in a Ca_v_2.1 channel-dependent depolarization in subsets of VTA neurons (Margolis et al., 2014, 2017). Further, the MOP agonist DAMGO increases Ca_v_2.1 currents in cerebellar Purkinje neurons (Iegorova et al., 2010) and morphine activates adenylyl cyclase in the corpus striatum and olfactory bulb (Puri et al., 1975; Onali and Olianas, 1991). While this is the first report of N/OFQ-mediated excitations in an acute brain slice preparation, intracellular increases in Ca^2+^ have been observed in a cultured human neuroblastoma cell line in response to N/OFQ in the presence of the cholinergic agonist carbachol (Connor et al., 1996). Therefore, while there are few reports of excitatory actions of N/OFQ, the observation is not unprecedented.

We also found that NOP activation signals through the canonical GIRK pathway in the SNc; however, in the VTA, N/OFQ outward currents were mediated by augmentation of GABA_A_R currents. This action via GABA_A_Rs is consistent with many of the N/OFQ-induced outward current sizes observed here being small, since our holding potential was only 10 mV depolarized from the calculated Cl^–^ reversal potential. We note that while our population sampling was conducted in p22–p35 rats (Fig. 1), our mechanism experiments were conducted in adults, raising the possibility that there might be some differences in the populations of neurons. We have previously found similar electrophysiological properties and dopamine D2 receptor and KOP receptor pharmacological responses in VTA neurons recorded from p35 compared with adult (>p60) rats (Margolis et al., 2008), and others have also reported mature firing patterns, biophysical properties, and dopamine D2 receptor GIRK-mediated responses in SNc dopamine neurons early in postnatal development (Tepper et al., 1990; Walsh et al., 1991; Wang and Pitts, 1995). Since the range of N/OFQ responses in the control population is relatively large, small changes in the distribution of N/OFQ responses because of the blockers would be difficult to detect statistically. That said, among SNc recordings, a small sample size with more homogeneous N/OFQ responses was sufficient to demonstrate a GIRK contribution to N/OFQ responses in adult rats. Thus, while age differences are a noted caveat regarding the statistical comparisons made here, prior studies show other GPCR responses in these neurons appear mature in p22–p35 rats.

While both GIRK activation and the GABA_A_R dependent mechanism generated outward currents in our experimental preparation, the physiological consequences of these neural populations using different signaling pathways *in vivo* may vary. For instance, activating a GIRK will always cause a hyperpolarization, while increasing the GABA_A_R conductance will only occur when there is concurrent activation of NOPs and GABA_A_Rs. Further, the N/OFQ-induced neural inhibition requiring GABA_A_R activation depends on the Cl^–^ reversal potential, which may be altered by a variety of behavioral states including pain, morphine treatment, stress, or alcohol exposure (Coull et al., 2003; Hewitt et al., 2009; Ferrini et al., 2013; Ostroumov et al., 2016; Santos et al., 2017). The N/OFQ response may even be excitatory in the absence of GABA_A_R activation, since blocking GABA_A_Rs seemed to increase the proportion of neurons in which we observed inward currents in response to N/OFQ (Fig. 5*C*).

In the VTA, neurons treated with a higher concentration of N/OFQ (≥100 nm) no longer responded to subsequent applications of N/OFQ in the VTA. This finding indicates that N/OFQ may act as a functional antagonist at the NOP by desensitizing these responses when higher concentrations of N/OFQ are present. Interestingly, we did not observe significant NOP desensitization in SNc neurons. NOP function is therefore apparently different from postsynaptic responses to agonists at the MOP and DOP in VTA neurons, where repeated application of saturating concentrations of selective agonists generate responses of similar magnitudes (Margolis et al., 2014, 2017). The apparent NOP desensitization we observed in the VTA is consistent with previous studies showing that high concentrations or repeated sustained exposure to NOP agonists causes desensitization in cell culture (Connor et al., 1996; Mandyam et al., 2000, 2002; Thakker and Standifer, 2002). In addition, NOPs internalize fairly rapidly (Spampinato et al., 2001, 2002; Corbani et al., 2004; Zhang et al., 2012) at the same concentrations that we observed desensitization. *In vivo*, N/OFQ administration can result in dose-dependent performance changes in behavioral spatial memory, locomotor, and anxiety tasks, with low-concentration N/OFQ having opposite effects compared with high doses (Florin et al., 1996; Jenck et al., 1997; Sandin et al., 2004). One possible explanation for these opposing behavioral outcomes is that N/OFQ may be acting as an agonist at low concentrations and a functional antagonist at high concentrations in some brain regions. An alternative possibility is that brain regions like the SNc that have less desensitization drive behavioral responses to high doses of N/OFQ, where brain regions like the VTA that show more desensitization mostly contribute to behavioral responses to lower N/OFQ doses.

N/OFQ inhibited both dopamine and non-dopamine neurons in the VTA that project to the NAc. This finding is consistent with the observation that N/OFQ administered ICV or into the VTA results in a decrease in extracellular dopamine in the NAc (Murphy et al., 1996; Murphy and Maidment, 1999). A prominent proposal in the literature is that a decrease in NAc dopamine produces aversion (McCutcheon et al., 2012). Therefore, one would expect ICV injection of N/OFQ to be aversive. However, this manipulation generates no response in the place conditioning paradigm (Devine et al., 1996). On the other hand, optogenetic or chemogenetic stimulation of N/OFQ containing inputs to the VTA can be aversive and decrease reward seeking (Parker et al., 2019). One possible explanation for this lack of clear motivational effect is the combination of inhibition of both dopamine and non-dopamine neurons: dopamine and non-dopamine neurons originating in the VTA synapse onto different types of neurons in the NAc, therefore affecting behavior in different ways. For instance, VTA glutamate neurons synapse onto parvalbumin containing interneurons in the NAc and optogenetic activation of these NAc-projecting glutamate neurons is aversive (Qi et al., 2016). Activation of NAc-projecting VTA GABA neurons causes a pause in cholinergic interneuron activity (Brown et al., 2012). These neurons modulate associative learning but are insufficient to drive preference or aversion independently (Collins et al., 2019) and do not appear to contribute to the detection of aversive gustatory stimuli (Robble et al., 2020). N/OFQ inhibition of dopamine, GABA, and glutamate neurons projecting to the NAc, therefore, may result in no net hedonic value and a lack of preference in a place preference paradigm. Further, various reports indicate that decreasing activity at dopamine receptors in the NAc via microinjections of antagonists does not produce aversion (Josselyn and Beninger, 1993; Baker et al., 1996, 1998; Morutto and Phillips, 1998; Laviolette and van der Kooy, 2003; Fenu et al., 2006; Spina et al., 2006; but see Shippenberg et al., 1991), and aversive outcomes can even be observed following manipulations that increase dopamine levels in the NAc (Devine et al., 1993b; Shippenberg and Bals-Kubik, 1995). Add to this the N/OFQ effects on other circuits following ICV injection, including other VTA neurons, and the possibility that the most robust, long-lasting effect is receptor desensitization at higher doses of agonist, together make it potentially less surprising that ICV N/OFQ was not reported to generate aversion.

N/OFQ’s effect on the VTA to NAc circuit provides an interesting point of comparison for how the NOP may be functionally distinct from the structurally related KOP. *In vivo*, systemic or ICV administration of N/OFQ or a KOP agonist each causes a decrease in extracellular dopamine in the NAc (Di Chiara and Imperato, 1988a,b; Devine et al., 1993a; Murphy et al., 1996; Murphy and Maidment, 1999). However, these two receptors function very differently in the dopamine neurons that project to the NAc. We show here that N/OFQ inhibits VTA cell bodies that project to the NAc but has little effect on the dopamine terminals within the NAc. KOP activation, on the other hand, has no effect on the cell bodies of NAc-projecting VTA dopamine neurons, but strongly inhibits dopamine release at the terminals in the NAc (Margolis et al., 2006; Britt and McGehee, 2008; Fig. 5*C*). One implication for this organization is that whether or not the respective endogenous peptides, N/OFQ and dynorphin, affect NAc-projecting dopamine neurons will depend on the brain region of peptide release. There is also evidence for dopamine release in the NAc that is independent of action potential firing in midbrain dopamine neurons (Cachope et al., 2012; Mohebi et al., 2019). In this organization of differential receptor effects localized to somadendritic regions versus terminals, dynorphin has control over this terminal activity while N/OFQ does not. Together, these observations bring into focus the critical importance of understanding precisely where receptors are functional in brain circuits and their specific actions at each site.

We found opposing effects of N/OFQ on the VTA projections to mPFC and pACC, which may contribute to the reported N/OFQ impact on behavioral measures associated with cortical dopamine function such as working memory, learning, and behavioral flexibility (Tzschentke, 2001; Winter et al., 2009; Gonzalez et al., 2014; Puig et al., 2014; Huang et al., 2019; Ott and Nieder, 2019). Our results also show that the non-dopamine VTA projections to cortical regions are affected by N/OFQ as well; while the majority of the VTA neurons that project to these cortical regions are not dopaminergic (Breton et al., 2019), little is currently known regarding their contribution to behavior. Preclinical studies show that ICV administration of N/OFQ impairs working memory (Hiramatsu and Inoue, 1999) and associative learning and memory (Goeldner et al., 2009), while blocking NOP with an antagonist or genetic knock-out enhances both working memory and learning (Noda et al., 1999; Jinsmaa et al., 2000; Nagai et al., 2007). How such a break on learning and memory by endogenous N/OFQ contributes to normal behavioral adaptation, and whether dopamine or other VTA outputs play a role, remains to be determined. One provocative possibility is that it is this degradation of working memory function that is the primary mechanism underlying the lack of place conditioning in response to central N/OFQ, rather than that this treatment is affectively neutral. This interpretation is consistent with work showing that N/OFQ blocks opioid induced conditioned place preference yet has no effect on opioid self-administration (Walker et al., 1998; Sakoori and Murphy, 2004).

The results of this study extend our understanding of the NOP system’s biology and provide considerations for additional investigation into NOP function within limbic circuits. These findings clarify that strong NOP desensitization occurs in neurons at moderate concentrations of the endogenous agonist N/OFQ. Importantly, not only does the nature of the NOP response vary with the projection target of VTA neurons, but the NOP function is largely sequestered to the somatodendritic compartment of VTA dopamine neurons that project to the NAc, demonstrating two different kinds of circuit level organization of this receptor system. Building on this groundwork, future studies of these VTA circuits during different behavioral states and tasks related to motivation and cognition will help to elucidate the differences between the normal and dysfunctional NOP-N/OFQ system, improving the potential for therapeutic targeting.

## Synthesis

Reviewing Editor: Douglas Bayliss, University of Virginia School of Medicine Decisions are customarily a result of the Reviewing Editor and the peer reviewers coming together and discussing their recommendations until a consensus is reached. When revisions are invited, a fact-based synthesis statement explaining their decision and outlining what is needed to prepare a revision will be listed below. The following reviewer(s) agreed to reveal their identity: Hugo Tejeda, Joseph Cheer.

Synthesis

This revised manuscript was seen by the original two reviewers, who generally approved of the responses and revisions in this submission. Nonetheless, some additional points require your attention. Most importantly, major concerns remain in the analysis and presentation of the data.

Specifically, the analyses of % responsive vs non-responsive cells remain unconvincing, a problem especially for cells in which currents induced by nociceptin/orphanin FQ (N/OFQ) were quite small (many <5 pA). Although a set of time-vehicle controls to establish a confidence interval for the background drift is still preferable, it was suggested in the consultation session that an alternative that does not require additional experiments is to provide, in extended data, multiple examples of “significantly responsive” cells from the lower end of response amplitude range (for both excitatory and inhibitory responses).

In addition, the unusual presentation of data in Fig. 5B,C is not ideal (violin vs. data points), and it is not clear from these data how one concludes that responses are preserved in BaCl2 (B), but eliminated in TTX, DNQX, and bicuculline (C) -- the sample sizes are small, the distributions largely overlapping, so the statistics that support these conclusions are not persuasive. Finally, it appears that the N/OFQ- Ba data were derived under different experimental conditions than the original N/OFQ data, making these comparisons even more suspect. The caveats associated with these comparisons (small samples, different conditions, etc.) must be made explicitly clear.

These and other concerns are detailed in the submitted reviews, which are appended below.

---

Reviewer #1

Advances the Field

An examination of the cellular effects of nociceptin will advance our understanding of the impact of the mechanisms by which this system regulates motivated behavior.

Statistics

The statistics used to determine whether responses were significant are inadequate and prone to detect spurious changes in holding current. The lack of an appropriate control and their lack of willingness to add control cells is concerning.

Comments to the Authors

The authors have addressed most of my comments in a satisfactory manner. However, a few issues remain that must be addressed to ensure that results are adequately interpreted.

Original Comment 3. The authors should provide more details on the pressure ejection system. Were there differences in drug responses when drugs were bath vs pressure applied? The authors should break down the analysis as the mode of application is predicted to affect things like desensitization and whether a cell is responsive or not depending if the pressure pipette covers the entire somatodendritic compartment of the cell.

Response from Authors. We appreciate these points raised by the reviewer and have added further information about the properties of the pressure ejection system in the methods section to address these concerns. For instance, the inner diameter of the ejection tip was 250 μm, which should cover the area containing the cell body and dendrites that we can monitor in voltage clamp (lines 126-128). We have also included example information, in the methods section, comparing the magnitudes of responses to 100 nM bath application and pressure ejection of N/OFQ (bath application 10.1 {plus minus} 1.5 pA, n = 21; pressure ejection 9.8 {plus minus} 2.1 pA, n = 12; lines 129-130). Finally, we have clarified that all of the experiments where repeated application of N/OFQ are reported were performed with bath application (lines 129 - 130).

From Current Review. I don’t see where they put the repeated application statement in lines 129-130

Original Comment 4. The authors should provide a better description of the mechanism by which N/OFQ hyperpolarizes and depolarizes neurons. It is assumed that this is GIRK-mediated via Gi/o proteins, but further evidence would be necessary to test the assumption. The authors should determine whether the putative inward currents are potentially a consequence of indirect circuit effects (i.e. inhibition of a tonic outward current). For instance, it would be useful to determine whether the inward current can be isolated in the presence of TTX and synaptic blockers (PTX, DNQX, AP-5) to block putative circuit effects. The authors should also determine whether inward currents are blocked by BTRX. If the inward current is truly found in ACC-projecting neurons then this neuronal population would be useful for the authors to concentrate on identifying the mechanisms that drive the inward current.

Response from Authors. We thank the reviewer very much for making this inquiry that significantly improved the content of this manuscript. In response to this inquiry we initiated a new set of experiments to (1) test whether we detected N/OFQ responses in the presence of synaptic blockers (TTX, DNQX, and bicuculline; now Fig. 5C) and (2) if we could block outward currents with BaCl2, to block K+ channel currents. All of these experiments were performed in tissue from adult rats. Surprisingly, we found that the synaptic blockers, but not BaCl2, prevented N/OFQ induced outward currents in the VTA (Fig. 5A-C). This raised additional questions of (1) why were our results different from the previous report that N/OFQ induced GIRK-mediated effects in VTA neurons (Zheng et al., 2002) and (2) what is the mechanism actually driving outward currents in VTA neurons? We conducted control experiments in SNc neurons, testing if, in our hands, BaCl2 would, in fact, block a N/OFQ response. In this way we confirmed that the BaCl2 “worked” (Fig. 5F, H). In this process, we also found much less desensitization in SNc neurons than we had seen in VTA neurons (Fig. 5G). Together, these observations are actually much more similar to the data in the Zheng et al 2002 report than our VTA recording data. We have also included a map of these recording sites (Fig. 5I) to very clearly show the difference between VTA and SNc recording locations in our report. As described in more detail in the revised manuscript, lines 293 - 297, for a variety of reasons we hypothesized that the actual mechanism underlying the N/OFQ induced outward currents in VTA neurons was through altering GABAAR mediated currents. In order to test this more directly, we utilized iontophoresis of GABA, isolating GABAAR responses with the GABABR blocker CGP35348. We found that N/OFQ (10 nM) increased the GABAAR mediated iontophoretic responses in some neurons, decreasing it in others, and these changes were directly related to the change in holding current induced by N/OFQ in the same neuron, suggesting that these responses are dependent upon alterations in tonic GABAAR mediated currents. While this is surprising, it is not completely unprecedented, as we have previously reported that activation of the delta opioid receptor can cause an increase in GABAAR mediated currents in VTA neurons from stressed rats. Together, these mechanism experiments have greatly expanded the findings of the manuscript and have enabled us to also expand our discussion section regarding the implications of these differences between the SNc and the VTA, and to discuss the possible functional implications for different inhibitory mechanisms of N/OFQ in these adjacent brain regions.

From Current Review. The Ba2+ effect is not clear or interpretable. These experiments were done in older animals and compared back to the previous dataset with young animals. Ideally, new control cells from the same animal would have been recorded to not only control for age but even time when the experiment was conducted. Since the original control data show many cells that at 10 nM show no obvious response, low sampling with Barium makes it hard to detect a difference. Even then, it appears that the response is smaller than what is shown in Fig 1, with really only one cell showing a response to 10 nM. The authors should also plot the 100 nM data. Were within-subjects experiments like those performed in SNc similarly performed for the VTA? That would be convincing to show that the established robust response is not Barium sensitive.

Original Comment 9. The criteria the authors used to determine whether a cell responded significantly with an inward or outward current is dangerous as it is prone to detect false positives. It assumes that drift in holding current would not be detected in the unpaired test. The authors should include vehicle control cells and determine whether the cells that are responsive to N/OFQ is due to a significant difference from vehicle (i.e. 2 SD cutoff for significance). Alternatively, the authors could apply their current criteria for significance to a large population of vehicle-treated cells and if none of the vehicle-treated cells are deemed significantly affected, then this would suggest the authors’ original strategy was okay. Moreover, on figure On Fig 3DE, there is a mismatch in the data points for the 10 nM.

Response from Authors. We thank the reviewer for these inquiries. We considered a variety of approaches to provide evidence that our statistical analyses are not generating false positive “response” classifications. We agree with the reviewer that any within cell statistical analysis can falsely classify rundown or drift as a “response”. We are careful to reject experiments with any drift in the baseline for this reason, but agree that additional evaluation would be helpful. First, we feel the statistical analysis we perform is appropriately powered: performing a power analysis in G*Power indicates that for a group size of 8, as used here, alpha error set at 0.05, power set to 0.95, and a standard deviation for each group of data points estimated at 3.4 pA (approximately that illustrated in the examples included in this manuscript), the minimum difference in means that is reliably detected as a significant change is 7 pA. We hope the time course data provided, both the individual examples and the group means, convinces the reviewer that no drift of this magnitude is contaminating our dataset. In Figure 4D that we show summary data for repeated responses of this magnitude in VTA neurons with 10 nM N/OFQ application. We also agree that providing additional systematic analysis on this point will improve the manuscript. We suggest that the 2 most helpful indicators that drift is not contributing to our “responses” are that the detection of the onset of changes are time locked to the onset of drug application, and that reversal of the changes are detected time locked to the wash out of the drug. For instance, if drift were a major factor, no washout reversal would be evident. To evaluate whether our classification approach is appropriate to detect these features, we performed a sliding window analysis, now included in an Extended Data document. Briefly, this analysis 4 shows both the time locked features of onset and reversal of drug effects are detectable.

From Current Review. While a power analysis and ensuring that baseline is as steady as possible is certainly appreciated, it does not truly address this reviewer’s concerns, particularly with cells with very small “responses”. Incorporation of cells lacking a “true effect” with cells with true responses in time courses would not necessarily reveal artifacts. The part where this is particularly concerning are for cells with extremely small “responses” (i.e. 5 pA or less). Drift would not have to be substantial in these cases. For instance, in Fig 5A, which does have a robust response, if we were to follow the baseline trend a drift of 5-10 pA would be apparent. Moreover, the mean data in fig 3B suggest that small drift is visible. Again, the present approach is likely incorporating cells with small drift and considering them responses.

Reviewer #2

Advances the Field

This revised manuscript provides interesting and novel findings of differential modulation of VTA circuits by the nociceptin/orphanin FQ system. Data show that mPFC and NAc-projecting VTA neurons respond to N/OFQ with outward currents whereas pACC-projecting VTA neurons respond with mostly inward currents. Furthermore, the authors reposrt that despite inhibitory effects at cell bodies, N/OFQ had little effect on stimulated dopamine release within the NAc as measured with FSCV.

Comments to the Authors

The authors have appropriately addressed my concerns. The manuscript is greatly improved, particularly the presentation of the methods and results, additional experiments have addressed the previous reviewer comments. The following revisions have adequately addressed previous concerns:

• Inclusion of extra controls and increased sample size in figure 7B (10 nM N/OFQ + light stimulation).

• Clarification of the numbers of animals used throughout and general improvement in the method section

• Addition of figures to show recording/electrode locations

Remaining comments:

Figure 5B - the comparison of the violin plot with the individual data is a little strange, and the inclusion of the single dark grey 100 nM condition is not clearly labelled on the plot (it just looks to be part of the 10 nM cluster). The authors could consider modifying this figure to be clearer.
